# Artificial intelligence enables scale, consistency, and rigor in forensic identity inference

**DOI:** 10.3325/cmj.2026.67.188

**Published:** 2026-06

**Authors:** Bruce Budowle, Stephen Newman, Nicole Jones, Misty Marra, Kristen Mittelman, David Mittelman

**Affiliations:** 1 Othram Inc, The Woodlands, TX, USA; 2 Department of Forensic Medicine, University of Helsinki, Finland; 3 RTI, Research Triangle Park, NC, USA

## Abstract

Advances in forensic genomics, which include massively parallel sequencing, dense single nucleotide polymorphism testing, and forensic genetic genealogy (FGG), have greatly expanded the range of cases in which DNA evidence can generate investigative leads. As a result, the primary limitations in modern forensic DNA analysis are no longer analytical sensitivity or marker availability, but the ability to reason consistently, transparently, and at scale over increasingly complex genetic, genealogical, and contextual information. Current forensic workflows remain predominantly human-centered, relying on manual reasoning that is difficult to standardize, reproduce, document, or scale across growing case inventories. The potential of artificial intelligence (AI) is described as an enabling layer for forensic identity inference, defined here as computational decision-support systems that structure, prioritize, and document reasoning over genetic associations, genealogical structures, and investigative context during identity hypothesis development. While formal statistical inference quantifies evidentiary weight, identity inference governs how evidence is explored, combined, and acted upon during investigations. AI-assisted systems can augment, but not replace, expert judgment by supporting scalable prioritization, relational reasoning, and systematic documentation of analytical decisions, while reducing bias. Properly designed AI-enabled systems offer a path to sustainably scaling FGG while supporting scientific rigor, accountability, and public trust.

Forensic DNA analysis has long been regarded as the gold standard of the forensic sciences, owing to its strong scientific foundations, extensive validation, and well-developed frameworks for interpretation and statistical evaluation (1-5). Traditional methods, including short tandem repeat (STR) typing and mitochondrial DNA analysis, have demonstrated high reliability when applied appropriately and have played a central role in forensic human identification. However, the designation of “gold standard” can obscure important limitations that have become increasingly apparent as the scope and scale of forensic applications expand.

Even within established DNA typing paradigms, substantial variability exists in analytical practices, interpretation strategies, and statistical reporting across and within laboratories and among practitioners (6,7). Differences in training, experience, institutional policies, and human judgment can yield subtle to divergent outcomes from similar data, occasionally resulting in inconsistent conclusions or errors (8,9). While expert oversight is essential, reliance on predominantly manual, human-centered workflows introduces unavoidable variation and limits the ability to scale forensic analysis in a consistent and reproducible manner.

## Human-centered interpretation and the limits of manual reasoning

Several factors contribute to differences in forensic DNA results and interpretation outcomes, two of which are particularly pervasive: the increasing volume and complexity of foundational and case-specific information, and the reliance on human-mediated interpretation. Achieving proficiency in the underlying theory and practical application of molecular biology, population genetics, statistics, casework considerations, and relevant legal requirements is challenging and, for many practitioners, overwhelming. To manage this complexity, policies are implemented often as substitutes for data-driven, case-specific judgment. However, such policy-based constraints can have unintended and, in some cases, serious consequences when they override analytical reasoning, as has been demonstrated in prior forensic review and oversight cases (10,11).

Additionally, wherever humans are involved in analytical processes – and they must be, particularly in interpretation and verification – variation in practice is inevitable. Skill sets differ, knowledge and experience vary widely, and individual confidence, heuristics, and cognitive biases influence decision-making. In the absence of strong unifying forces, consensus on analytical procedures is difficult to achieve, even for seemingly minor aspects of interpretation, as demonstrated in inter-laboratory and round-robin studies (12-16).

Compounding these challenges is the siloing of expertise within the forensic community. Deep technical understanding, process improvements, and effective problem-solving and bias reduction strategies often reside within a small number of individuals and are not systematically captured, shared, or reused. As a result, knowledge and experience do not compound across cases, individuals, or organizations, and improvements are not realized dynamically or effectively at the field level. These limitations cannot be addressed through training, staffing, or policy refinement alone, as they arise from structural and human-bias constraints on how reasoning is performed, preserved, and reused. Despite long-standing awareness of these issues, there have been relatively few efforts to move beyond traditional, manual-centric approaches in forensic DNA analysis.

## The expansion of forensic genomics and the new bottleneck

Over the past decade, forensic DNA analysis has undergone a profound transformation. Advances in massively parallel sequencing (MPS), the adoption of dense single nucleotide polymorphism (SNP) testing, and the emergence of forensic genetic genealogy (FGG) have expanded the range of forensic biological evidence that can be analyzed and the degree of kinship that can be inferred (17-24). These developments have enabled investigative leads from highly degraded or low-quantity biological samples, distant genetic relationships, and cases that historically produced little or no actionable information. Dense SNP testing and genome-wide sequencing have substantially reduced many technical barriers that once limited obtaining results from forensic DNA analysis (17,18,21,25,26).

As a consequence of these advances, analytical sensitivity and marker density are no longer the dominant limitations in contemporary forensic genetics; instead, the central challenge is the capacity to reason over complex relational information at scale. Each FGG case may generate dozens to hundreds of potential genetic associations, genealogical pathways, and contextual leads, many of which must be evaluated, prioritized, and either pursued or excluded (27-29). When this reasoning is performed manually, success depends heavily on individual expertise, time availability, and institutional resources. Analytical strategies are rarely formalized, lessons learned in one case are not systematically transferred to others, and reasoning often resides in the tacit knowledge of a small number of practitioners. As case volumes increase and more agencies implement FGG, this model leads to delays, inconsistent outcomes, underutilization of available data, and large backlogs of cases that may never be fully explored despite being technologically solvable.

Artificial intelligence (AI) and related computational approaches offer a means to address this structural mismatch between the power of modern forensic DNA technologies and the workflows used to operationalize them. When appropriately designed and governed, AI-enabled systems can augment forensic workflows by supporting scalable reasoning, prioritization, and documentation under expert supervision. In the context of FGG, AI has particular relevance due to the inherently relational nature of genealogical inference (30), the need to evaluate numerous competing hypotheses (29,31), and the importance of preserving transparency, auditability, and accountability in investigative decision-making. This article describes the role of AI as an enabling layer for modern forensic DNA analysis, focusing on ways AI-supported systems can operationalize forensic reasoning at scale while remaining scientifically rigorous, scientifically supportable, and ethically responsible (32).

## The evolution of forensic DNA analysis workflows

Historically, the evolution of forensic DNA analysis has been driven by advances in laboratory technology and molecular biology. Early efforts focused on developing robust marker systems and analytical methods capable of producing reliable results from biological evidence. Eventually, these efforts led to widespread adoption of STR typing using capillary electrophoresis (CE), with the vast majority of forensic DNA laboratories worldwide converging on a modest number of standardized STR markers (33-35). Each transition, from variable number tandem repeats and restriction fragment length polymorphism approaches (36-38) to STRs, and more recently to SNP-based methods and MPS, expanded the range, quantity, and quality of biological samples that could be analyzed and improved the resolution in which individuals could be distinguished or biological relationships inferred.

While these technological advances substantially increased data generation capabilities, the interpretive workflows used to analyze forensic DNA results are evolving more slowly. Initially, manual interpretation and case-by-case expert reasoning were sufficient to support routine forensic casework. Analytical processes remained largely human-centered, reflecting practices developed at a time when the number of actionable leads generated from DNA evidence was comparatively limited and computational resources were modest.

As analytical complexity increased, computational assistance was introduced selectively to address specific challenges. Probabilistic genotyping systems were developed to formalize expert reasoning and extend the interpretability of complex DNA mixtures and lower-quality profiles by applying statistical models to evaluate competing hypotheses under uncertainty (39,40). Similarly, computational approaches enabled the prediction of outwardly visible phenotypic features from genetic data (41,42). These tools demonstrated that computational methods could improve consistency, reproducibility, and transparency while preserving expert oversight. They also marked an important shift toward integrating computation into forensic interpretation where analytical demands exceeded the practical limits of manual workflows.

The adoption of dense SNP testing and the emergence of FGG introduced a qualitatively different analytical challenge. SNP-based profiling and genealogical database searching can generate numerous potential genetic associations in a single case, particularly when distant relationships are considered. Interpreting these results requires integrating genetic data with genealogical records, public data sources, and contextual information, often across large and complex family networks (31,43). These tasks differ fundamentally from earlier forensic DNA analyses, which involved evaluating typically two or a very limited number of hypotheses and often considered only one or two potential contributors.

As FGG became more widely applied, the nature of forensic DNA workflows shifted from primarily evaluating isolated genetic comparisons to reasoning across relational and genealogical structures. This transition required overcoming limitations in workflows that were not designed to support systematic prioritization, hypothesis testing, or documentation across large sets of interconnected leads.

## Artificial intelligence, machine learning, and inference in forensic contexts

Methods in AI, machine learning (ML) and other computational approaches have been explored in forensic genetics primarily as tools to assist with the interpretation of DNA data (44-50). Some examples of such work have examined computational methods for tasks such as mixture deconvolution (ie, probabilistic genotyping), electropherogram interpretation, prediction of outwardly visible phenotypic traits, and the classification of Y-chromosome and population haplogroups from STR or SNP data. While these applications demonstrate the ability of computational systems to manage uncertainty and complex genetic data structures, they largely function as localized classification or interpretation tools rather than as components of an integrated forensic reasoning system capable of supporting broader investigative inference.

Instead, AI can be used in a functional sense to describe computational decision-support systems that structure, prioritize, and document forensic reasoning over complex, relational information under human supervision. This definition distinguishes AI-assisted inference from autonomous predictive modeling and emphasizes continuity with established forensic computational approaches, such as probabilistic genotyping and Bayesian reasoning. The primary contribution of AI in this context is scalable coordination, transparency, and reproducibility of investigative reasoning.

A notable gap in the application of AI and ML in forensic DNA analysis has been the limited attention given to reasoning over non-genetic and contextual information. While computational tools have been used to evaluate genetic evidence itself, comparatively little effort has been devoted to assisting with the integration, prioritization, and evaluation of metadata that contribute to forensic intelligence regarding genealogical records, public data sources, case context, and investigative constraints. One exception is the use of structured probabilistic frameworks, such as Bayesian networks applied to activity-level propositions. They provide formal reasoning models (51,52) but remain limited in adoption and scope within routine forensic practice and have met some resistance in application (53,54).

AI-enabled approaches are not a conceptual departure from established forensic methods but rather an extension of prior computational reasoning practices. The adoption of probabilistic genotyping and related systems demonstrated that formalized computational models could improve data acquisition and interpretation when analytical complexity exceeds the practical limits of manual workflows. Modern AI systems build on this foundation by extending computational support beyond isolated analytical tasks to encompass prioritization, relational inference, and structured reasoning across interconnected data.

In forensic science, the term “inference” is used often to describe formal statistical inference, such as the evaluation of competing propositions using likelihood ratios or probabilistic models. Statistical inference plays a central role in assessing the strength of specific evidentiary propositions and is supported by well-established mathematical frameworks (44). However, forensic investigations, particularly those involving FGG, rely on a broader form of identity inference. Identity inference encompasses the reasoning processes used to prioritize leads, evaluate alternative genealogical hypotheses, integrate genetic findings with contextual information, and determine which analytical paths warrant further exploration, in pursuit of an identification. While statistical inference addresses the weight of particular evidence, identity inference governs how evidence is explored, combined, and acted upon to yield an identity hypothesis.

AI encompasses computational methods designed to support pattern recognition and inference in the presence of uncertainty, incomplete information, and noise. In forensic contexts, the value of these approaches lies in augmenting human reasoning by managing scale and complexity through organizing information, surfacing relevant associations, and supporting systematic evaluation of competing investigative hypotheses.

Identity inference is the operational phase of forensic analysis that carries legal, ethical, and societal consequences, as it directly influences investigative direction and may be subject to judicial scrutiny. Accordingly, AI-assisted forensic systems must prioritize transparency, reliability, and auditability of inference processes rather than focusing solely on performance.

## Human-centered forensic genetic genealogy: strengths and limitations

Human expertise remains central to FGG. Experienced analysts bring contextual understanding, domain knowledge, and ethical judgment essential for responsible casework. Manual genealogical analysis requires investigators to resolve ambiguities in historical records, weigh nuanced factors, and incorporate case-specific information that may not be captured in structured data sources. These elements have contributed to the successful resolution of many cases but can quickly become overwhelming as case complexity increases.

Much of the reasoning involved in genealogical analysis remains implicit and is rarely captured in a systematic or reusable form. To scale effectively, successful analytical strategies must translate across cases, analysts, and organizations, support continuous improvement, and remain auditable.

The expansion of FGG is constrained by limited scalability and sustainability of FGG workflows. One such limitation arises from the heterogeneity of cases. Investigations vary widely in complexity, ranging from cases resolved through direct kinship testing or database searches with close genetic relatives, to cases involving distant relatives, sparse records, and complex family structures. Treating all cases as analytically equivalent in terms of expected complexity, evidentiary value, and investigative strategy can lead to inefficient allocation of resources, non-productive outcomes, and prolonged casework timelines.

Another structural limitation stems from historical reliance on analytical tools developed for consumer genetic genealogy rather than for forensic applications. While these tools facilitated early adoption of FGG, they were not designed to accommodate the variable quality of forensic DNA profiles or the governance requirements associated with criminal investigations. Dependence on externally governed platforms introduces operational vulnerability, as access to essential analytical capabilities may be constrained by policies or decisions outside the control of forensic practitioners, and can increase risks related to data security and privacy (31,55,56).

An additional challenge is prioritization, as the number of FGG-eligible cases continues to grow. Determining which cases are most likely to yield results with a given level of effort is often based on manual assessment, expert judgment, or local policy.

Genealogical analysis involves a series of decisions regarding the genetic relationships that are to be prioritized, which family branches to explore, and which hypotheses to pursue or exclude. When this reasoning is not systematically documented, successful outcomes do not readily translate into shared knowledge or improved practices across cases or organizations, and do not lend themselves well to critical review. Addressing these structural constraints requires workflows capable of supporting prioritization, reducing unnecessary variability, and enabling consistent, transparent analysis at scale without compromising scientific rigor.

## Artificial intelligence as an enabling layer in forensic investigations

AI in forensic investigations should be considered an enabling layer that augments existing scientific methods. In FGG, AI does not function as a standalone solution; instead, it supports coordination across analytical stages that together contribute to identity inference. Genome sequencing, database searching, genealogical reconstruction, and contextual investigation each provide partial context, and identification emerges only when these components operate as a coherent inference system. In this role, AI systems do not generate identifications or evidentiary conclusions, but support human experts by organizing information, prioritizing investigative paths, and preserving transparent decision traces for review.

AI-enabled workflows contribute to consistent information flows across stages of analysis, allowing improvements in one component to propagate throughout the investigative process rather than remaining isolated. Automation also supports transparency by enabling systematic documentation of intermediate steps, decision criteria, and alternative hypotheses considered during analysis. Preserving these decision traces strengthens quality assurance, facilitates peer review, and supports judicial scrutiny. Therefore, AI extends established forensic practices by enabling consistent, auditable reasoning at scale rather than introducing a new investigative paradigm.

## Artificial intelligence-enabled automation in forensic genetic genealogy workflows

Once forensic SNP profiles are generated and database searches completed, investigators are often presented with numerous genetic relatives (sometimes referred to as “genetic matches”) of varying relevance for establishing an identity hypothesis. Manually evaluating these results can be time-consuming and inconsistent, particularly as case volumes increase.

Although genetic genealogy practitioners often refer to the results of database searches as a match list, the term “match” is ambiguous and potentially misleading in a forensic context. In traditional forensic disciplines, “match” has been used to imply a direct correspondence between evidentiary material and a known source, and with DNA typing has been restricted to describing essentially single-source profiles. In contrast, genome-wide SNP testing and FGG typically do not produce direct source matches but instead identify associations with putative genetic relatives. These associations represent hypotheses about shared ancestry rather than direct identifications. To reduce ambiguity and better reflect the underlying science when describing the results of FGG database searches, the forensic field may benefit from adopting terminology such as genetic relative, genetic relationship, association or other similar conceptual wording rather than match.

AI-assisted automation can structure the identity inference process by supporting prioritization and triage based on measurable case characteristics. Cases can be stratified according to the quantity and quality of the DNA extracted from a sample, the number and strength of associations of genetic relatives, clustering patterns, availability of reference samples, and other relevant indicators. Thus, analytical efforts can be aligned with the likelihood of resolution, so that simpler cases are addressed efficiently while more complex cases receive appropriate attention.

Automation also can assist genealogical analysis by systematically integrating genetic relationship data with genealogical records. Graph-based representations of family relationships allow computational tools to more efficiently surface potential connections, identify shared ancestors, and highlight areas of interest within large pedigrees. In addition to reducing the manual effort required to explore complex family networks, these approaches can improve consistency across cases. By applying analytical rules and prioritization criteria uniformly, AI-enabled workflows reduce unnecessary variability and support consistent documentation of investigative reasoning..

## Moving to a fit-for-purpose, AI-enabled system for forensic genetic genealogy

Both an advantage and a major challenge of FGG is the vast amount of data generated during analysis. As data volume and complexity increase, the human interface becomes a bottleneck. These challenges are amplified by the characteristics of forensic DNA evidence. Unlike consumer DNA profiles, forensic profiles often originate from degraded, limited quantity, or environmentally compromised samples. As a result, they may not present a complete marker profile, contain elevated noise, and/or include erroneous SNP calls, making it difficult to distinguish meaningful genetic relationships from spurious associations. When such profiles are processed using disjointed tools, separating laboratory analysis, genomic interpretation, genealogical reasoning, and contextual investigation, there is an increased likelihood of inefficiency, missed connections, and analytical failure.

Addressing these limitations requires complex coordination across the entire identity inference process. Rather than optimizing individual steps in isolation, FGG must be treated as a linked system that spans biological evidence digitization, preparation of inference-ready genomic signals, and coordinated reasoning over genetic and contextual information. Optimizing each step with feedback improvement within a unified framework reduces manual, error-prone data handling, improves resource allocation, and enables timely and supportable outcomes. AI-enabled systems are particularly well suited to support such coordination by providing the speed, scale, and consistency necessary to manage complex inference while preserving expert oversight. Importantly, such systems allow experience gained from individual investigations to be captured and reused, rather than remaining confined to the tacit knowledge of a small number of practitioners.

## Architecture of the artificial intelligence-assisted identity inference system

The AI-assisted FGG system recently built by Othram Inc. (The Woodlands, TX, USA) was constructed as a coordinated, hypothesis-directed identity inference framework designed to operationalize genealogical reasoning under forensic constraints. Rather than functioning as a general-purpose generative model, the system operates within a structured, tool-mediated orchestration environment in which specialized computational agents execute defined analytical tasks under human supervision. Its primary objectives are to contextualize genetic relatives identified through SNP-based database searches and to expand genealogical structures in the direction of an unknown individual.

AI agents access controlled tools, including web search, structured document retrieval and parsing, optical character recognition, and querying of publicly available historical data sets. Analytical context derived from the evolving genealogical graph, such as names, dates, locations, and known relationships, is used to evaluate newly retrieved sources. When corroboration criteria are met, evidence is attached to relevant nodes within a graph-based evidence system, and a decision trace is generated documenting the source, matching rationale, and resulting structural updates. This process transforms implicit genealogical reasoning into explicit, inspectable analytical artifacts.

Because FGG cases often begin with numerous putative genetic relatives, the system initiates parallel tree construction processes and continuously evaluates overlap among independently expanded branches. Convergent ancestral nodes are identified and merged within the graph framework, generating identity hypotheses consistent with aggregate genetic associations. Throughout the process, human analysts retain authority to review and refine system outputs, and all analytical actions are preserved for auditability.

## An integrated reference implementation for coordinated identity inference

One example of a fit-for-purpose system designed specifically to support coordinated identity inference in FGG is the Multi-Dimensional Forensic Intelligence (MDFI) platform (57). MDFI integrates laboratory outputs, genomic signal preparation, genealogical reasoning, and investigative coordination into a single, documented analytical environment. This integration allows data, intermediate decisions, and conclusions to be traced, assessed, and reviewed throughout the inference process, supporting quality assurance, continuous improvement, transparency, and judicial scrutiny.

MDFI is composed of coordinated components aligned with distinct stages of identity inference. Forensic-Grade Genome Sequencing® (FGGS®) addresses the digitization of physical biological evidence. SNPSuite prepares inference-ready genomic signals by transforming raw sequencing output into standardized, quality-controlled representations suitable for kinship analysis. KinSNP® supports kinship inference by measuring identity-by-descent (IBD) segment sharing across dense SNP data. Othram Maps provides the graph-based reasoning environment in which genetic signals, genealogical structures, and public-record context are integrated to develop and evaluate identity hypotheses. OthramOS preserves investigative context, reasoning artifacts, and institutional knowledge as cases scale and teams change (58).

Presented here as a reference implementation, MDFI is used to illustrate how purpose-built components can be coordinated to preserve inferential signal, reduce manual burden, and maintain coherence across the identity inference pipeline. While MDFI is used as an example, the architectural principles described are general and could be realized using other purpose-built systems designed around coordinated identity inference.

## Identity inference as a coordinated three-stage pipeline

**Figure 1:**
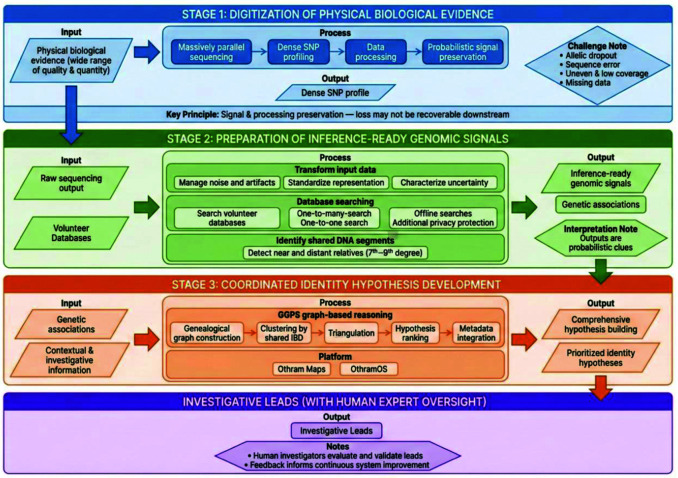
**The coordinated identity inference workflow in forensic genetic genealogy (FGG).** The diagram illustrates the flow of information from biological evidence through genomic processing and relational analysis to prioritized identity hypotheses. Arrows indicate directional data transformation across stages, with outputs from each stage serving as inputs to the next stage. The final enabling layer highlights human expert oversight and feedback, emphasizing that investigative leads are generated within a documented and auditable analytical framework. The Genetic Genealogical Positioning System (GGPS) graph-based reasoning system is described in the section titled Stage 3: Coordinated identity hypothesis development.

Identity inference in FGG consists of three basic stages: (i) digitization of physical biological evidence, (ii) preparation of inference-ready genomic signals, and (iii) coordinated development of identity hypotheses using genetic signals and contextual information (Figure 1). Each stage transforms information in ways that constrain downstream inference. Signal loss, distortion, or fragmentation introduced upstream cannot be reliably corrected later, making coordination and purpose-built optimization essential.

### Stage 1: Digitization of physical biological evidence

The first stage of identity inference involves generating a dense SNP profile from biological evidence, typically through genome-wide, MPS. In forensic contexts, this step differs fundamentally from genetic testing in research, consumer, or even medical contexts. Forensic samples are often degraded, fragmented, compromised by environmental insults, and limited in quantity. These properties introduce downstream typing and interpretive challenges such as allelic dropout, damage-induced errors, and uneven and low coverage/read depth.

Digitization methods optimized for high-quality and high-quantity DNA samples are poorly suited to these conditions and may discard weak but informative signals or amplify noise in ways that are difficult to detect downstream. Because all subsequent inference operates on digital representations of biological evidence, information lost or distorted at this stage may not be reliably recovered downstream. Inferential signal and uncertainty may be altered or lost if data are not consistently preserved and transparently transferred between analytical stages. Manual file handling, format conversion, or ad hoc filtering decisions, often applied to manage noise or incomplete data, can remove weak but informative genetic signals or obscure their provenance. Once such signal is lost at the digitization stage, it may not be reliably recovered later, even if subsequent analytical or genealogical steps are well executed.

Importantly, determining which signals are informative at the digitization stage cannot be done reliably in process-step isolation. The relevance of low-coverage or noisy genetic information depends on its downstream contribution to kinship detection, genetic relationship convergence, and hypothesis evaluation, which are outcomes that are not observable at the time of data generation. As a result, optimization decisions made during digitization without feedback from downstream inference risk discarding information that would later prove critical.

Forensic-grade digitization therefore prioritizes sensitivity, damage awareness, and preservation of probabilistic signal rather than completeness or convenience. The goal is to maximize the inferential value of imperfect biological evidence in a manner compatible with coordinated signal preparation and identity hypothesis development.

### Stage 2: Preparation of inference-ready genomic signals

Raw sequence output, even when generated using forensic methods, is not directly suitable for identity inference. The second stage transforms raw digital data into inference-ready genomic signals that support probabilistic and relational reasoning. This process includes managing noise and artifacts, standardizing representation across cases, characterizing uncertainty, and preparing signals compatible with kinship inference.

Within this stage, dense SNP profiles derived from evidence are searched against databases containing profiles from individuals who have voluntarily, with informed consent, donated their DNA to assist law-enforcement investigations. Associations between near and distant relatives are identified based on shared DNA segments measured in centimorgans, with greater shared DNA indicating closer kinship relationships. Using comprehensive SNP data, associations can extend to seventh- to ninth-degree relatives (59-62).

Each genetic association represents a probabilistic clue rather than a direct identification. Importantly, generic or non-forensic preprocessing pipelines often emphasize metrics such as call rate or completeness that are poorly aligned with the needs of FGG. Such approaches can distort relational signal, reduce sensitivity to distant kinship, or increase susceptibility to background genetic noise. Inference-ready signal preparation instead emphasizes consistency, interpretability, and preservation of weak but meaningful genetic associations, facilitating downstream reasoning to operate on comparable and reliable signals.

### Stage 3: Coordinated identity hypothesis development

The final stage of identity inference integrates inference-ready genetic signals with genealogical structure, public records, and investigative context to generate, evaluate, and refine identity hypotheses. Each genetic association represents a potential path toward the unknown individual at the center of an investigation. The challenge lies in prioritizing these paths by identifying those most likely to converge on a common ancestor to connect to the unknown individual, while deprioritizing or excluding paths that are unlikely to yield productive identity hypotheses.

**Figure 2:**
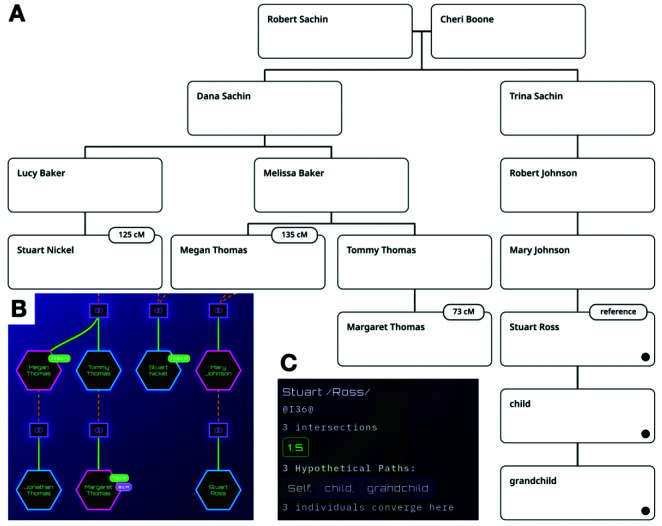
**MRCA inference and Genetic Genealogical Positioning System (GGPS)-guided reference selection in Othram Maps.** (**A**) Auto-generated most recent common ancestor (MRCA) plot showing individuals who share DNA with the unknown profile (cM values shown) and the inferred position of the unknown (“reference”); black circles mark plausible reference candidates. (**B**) A corresponding interactive pedigree view in Othram Maps integrating autosomal and X-chromosome DNA to constrain genealogical paths. (**C**) GGPS output indicating convergence of three intersection signals on the Stuart Ross lineage, prioritizing Stuart Ross, or his child or grandchild, for confirmatory reference testing. The names and relationships displayed are fictitious and do not represent any real individuals. All three panels are direct screenshots or system outputs from Othram Maps.

An illustrative example of coordinated identity hypothesis development is the Genetic Genealogical Positioning System (GGPS), a graph-based analytical framework designed to support FGG by automating the generation, ranking, and visualization of genealogical hypotheses from DNA association data (43). Rather than treating genetic associations as isolated events, GGPS systematically explores all plausible ancestral paths linking an unknown individual to known relatives within a genealogical graph, quantifies both the evidentiary support and expected investigative effort associated with each path, and prioritizes hypotheses most consistent with the observed genetic evidence (Figure 2). In doing so, GGPS operationalizes the process of genealogical reasoning that is traditionally performed manually by expert practitioners. Reasoning is transformed into a structured, reproducible, and reviewable identity-positioning process. By making hypothesis generation and prioritization explicit rather than implicit, GGPS illustrates how coordinated, AI-enabled systems can preserve expert judgment while enabling consistent reasoning, auditability, and governance at scale.

Genealogical analysis is inherently relational. Family trees consist of individuals connected through parent-child and spousal relationships, forming complex networks that expand substantially across generations. As the number of potential relatives increases, particularly when distant kinship is involved, these networks become difficult to navigate using linear or ad hoc methods.

Graph-based models provide a natural framework for representing and analyzing genealogical relationships. Individuals are represented as nodes, familial relationships as edges, and genetic associations as attributes. In Othram Maps, genealogical data commonly exchanged in GEDCOM (GEnealogical Data COMmunication) format (63) are rendered as living graphs, enabling systematic traversal, detection of redundancies such as pedigree collapse, and layering of additional evidence. Hypotheses can be tested, DNA match data overlaid, and lineage extension or consolidation automated within a structured and auditable framework.

### Hypothesis reduction through clustering

Searches may yield a few or many putative kinship associations, some reflecting true shared ancestry and others arising from background genetic noise or limitations in profile quality. Clustering group associations based on the amount and genomic location of shared IBD segments focuses analyses on the most informative family branches. These clusters often correspond to distinct ancestral lines and substantially reduce the hypothesis space that must be explored manually. Additional features, such as assessment of specific shared segments, biogeographical ancestry, and surnames, further refine analysis and help investigators focus on directly connected DNA associations. Automated clustering reduces target-driven perception bias by identifying meaningful genetic relationship patterns before human interpretation.

### Evidence strengthening through triangulation

Once clusters are identified, triangulation provides a method for strengthening evidence by verifying that specific DNA segments are shared by three or more individuals at the same genomic location. This process confirms that shared DNA reflects inheritance from a common ancestor rather than by chance. Triangulation enables more precise lineage reconstruction, particularly when dealing with distant relatives or incomplete profiles, and supports elimination or prioritization of hypotheses.

## Integration of investigative metadata

**Figure 3:**
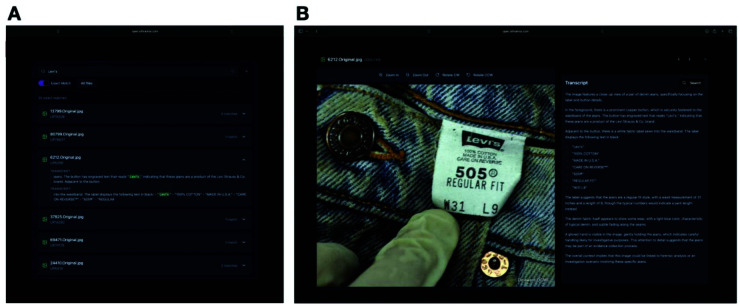
Artificial intelligence (AI)-assisted extraction of image-derived investigative intelligence in NamUs case UP6288. (**A**) Structured search results in OthramOS showing that a query for “Levi’s” returns case UP6288, even though the publicly available NamUs case record does not explicitly mention the brand. (**B**) Source image displaying a Levi’s label and the corresponding AI-generated transcription and semantic analysis. Automated extraction of visual text enabled the creation of searchable metadata linked to the case, illustrating how multimodal AI can integrate unstructured evidence into investigative and genetic reasoning workflows.

Genetic inference alone may be insufficient to resolve identity. Genetic hypotheses are validated, refuted, or refined through investigative intelligence (timelines, witness statements, geospatial information, digital evidence, and public records). However, the volume and heterogeneity of such data make manual integration difficult and inconsistent. AI-enabled systems allow disparate data sources (paper records, audio, video, and images) to be digitized, transcribed, translated, and organized into structured, searchable formats (Figure 3). Linking these data to genetic and genealogical reasoning enables investigators to connect critical details more efficiently and generate investigative leads with greater precision.

## Objectivity, auditability, and continuous improvement

Automated and AI-assisted workflows reduce unintended variability by applying consistent analytical criteria across cases. Explicitly encoded prioritization rules, filtering thresholds, and hypothesis-evaluation procedures support objectivity while preserving expert judgment.

Automation also improves auditability by documenting structured records of analytical reasoning. These decision traces capture intermediate steps, assumptions, exclusions, and alternative hypotheses, transforming implicit expert reasoning into inspectable and accountable analytical artifacts. When captured consistently across cases, decision traces enable continuous improvement by revealing which strategies are effective and where inefficiencies arise. Importantly, this learning is grounded in documented human reasoning rather than autonomous model updating, allowing systems to scale while continuously improving effectiveness and efficiency.

## Governance and ethical safeguards

The incorporation of AI into FGG requires governance mechanisms that ensure transparency, accountability, privacy protection, and human oversight. Integrated systems support these goals by enforcing access controls, standardizing documentation, and maintaining detailed audit trails of analytical actions. Minimizing unnecessary third-party access reduces privacy risks, particularly for individuals who are not suspects but whose data may be associated through genetic relationships (31).

AI-assisted workflows also introduce risks such as automation bias and overreliance on computational outputs. While AI cannot fully eliminate bias, it introduces auditable bias sources that can be systematically monitored and addressed. Effective governance therefore requires explicit documentation of where expert judgment intervenes, overrides, or refines automated results. Analytical methods must remain explainable, and investigative leads must be traceable to underlying data and defined reasoning steps (64).

AI-based systems can operationalize transparency through several mechanisms. Graph-based genealogical reasoning provides inherent explainability: each identity hypothesis is represented as a traversable path through a family network, with evidentiary support quantified at each node. Platforms can preserve complete decision traces documenting genetic associations evaluated, clustering parameters applied, triangulation results, and the rationale for prioritizing or excluding specific genealogical branches. A critical requirement of scientific validity is reproducibility. Given the same genetic data and genealogical inputs, AI-driven tools should yield similar hypothesis rankings. Ultimately, scalable and supportable FGG inferences depend on systems that are technologically capable as well as transparent, properly governed, and aligned with forensic and societal expectations. Purpose-built, coordinated identity inference systems provide a path to expanding the impact of FGG while maintaining scientific rigor, accountability, and public trust.

## Concluding remarks

Advances in forensic DNA technology continue to transform the ability of investigators to generate leads from biological evidence. Dense SNP testing, genome-wide sequencing, and FGG have expanded the scope of cases in which DNA can contribute meaningfully to investigations, including analysis of highly degraded samples, distant kinship associations, and long-unsolved crimes. As these methods have matured, the central challenge of forensic DNA analysis has shifted from data generation to consistent interpretation, prioritization, and operational use of increasingly complex information at scale.

Viewed in context with previous computational developments in forensic genetics, AI represents a natural extension of computational assistance aimed at managing complexity and uncertainty. By augmenting forensic workflows with automation, structured reasoning, and scalable analytical support, AI can help improve consistency, transparency, and efficiency in the application of FGG (Table 1).

**Table 1:** Analytical domains in forensic genetic genealogy (FGG), corresponding contrasts between predominantly manual and artificial intelligence (AI)-enabled workflows, and impact of AI-enabled workflows. Key components of FGG casework are organized, including triage, genetic association interpretation, genealogical hypothesis development, contextual integration, documentation, and scalability. For each domain, the operational implications of augmentation across investigative and analytical processes are outlined

Analytical domain	Predominantly manual approach	AI-enabled augmentation	Primary impact
Case prioritization and triage	Expert-driven case selection based on qualitative review	Structured scoring using measurable case features (DNA quality, genetic relative strength, clustering patterns, reference availability)	Improved resource allocation and consistent prioritization
Genetic association interpretation	Manual review of genetic relatives; subjective selection of branches	Automated clustering, segment-based grouping, and relevance ranking	Reduced cognitive bias load; improved consistency in identifying informative lineages
Genealogical hypothesis development	Linear, analyst-driven tree building and hypothesis exploration	Graph-based reasoning, automated path enumeration, and hypothesis ranking	Scalable exploration of complex pedigrees with transparent prioritization
Integration of contextual information	Manual synthesis of genealogical records, timelines, geospatial and investigative metadata	Structured ingestion and linkage of heterogeneous data to relational graphs	More systematic cross-domain reasoning; reduced missed connections
Documentation and auditability	Narrative case notes with partial capture of reasoning	Automated decision traces preserving parameters, exclusions, and alternative hypotheses	Enhanced transparency, reproducibility, and judicial defensibility
Scalability and knowledge reuse	Analyst-limited throughput; tacit expertise	Encoded analytical frameworks reusable across cases	Sustainable scaling of FGG while preserving scientific rigor

These capabilities are particularly important as jurisdictions address large inventories of unsolved violent crimes and unidentified human remains, where sustained, high throughput, yet scientifically supportable, approaches are required. When deployed within appropriately governed, fit-for-purpose systems, AI can support the sustainable scaling of FGG while maintaining scientific rigor, transparency, and legal scrutiny.

AI-assisted genealogical inference does not imply autonomous decision-making. The role of AI in this context is not to replace human expertise or established forensic principles, but to operationalize them more effectively. By formalizing elements of expert reasoning and preserving them as explicit, auditable analytical artifacts, AI-enabled systems can support systematic exploration of complex genealogical relationships, facilitate collaboration across analysts and organizations, reduce unnecessary variability in investigative workflows, and importantly, better capture the untapped wealth of knowledge and expertise of the discipline.

As forensic science continues to evolve, the integration of AI should be understood as an enabling infrastructure rather than a paradigm shift in decision authority. By focusing on scale, consistency, documentation, and accountability, AI-enabled systems have the potential to fully realize the benefits of modern forensic DNA technologies in a responsible manner. In doing so, there will be greater support for public safety, judicial scrutiny, and the resolution of cases that have remained unanswered not because of technological limitations, but because of the challenges inherent in managing complex inference at scale.
